# Factors Associated With Readmission to Index vs. Non‐Index Hospitals After Major Cancer Surgery

**DOI:** 10.1002/cam4.71359

**Published:** 2025-12-17

**Authors:** Avanish Madhavaram, Shan Wu, Danielle R. Sharbaugh, Yuanbo Zhang, Jonathan G. Yabes, Kathryn A. Marchetti, Michael G. Stencel, Kimberly J. Rak, John A. Lech, Emilia J. Diego, Pascal O. Zinn, Sarah Taylor, Rajeev Dhupar, Dana H. Bovbjerg, Tiffany L. Gary‐Webb, Jeremy M. Kahn, Lindsay M. Sabik, Bruce L. Jacobs

**Affiliations:** ^1^ University of Pittsburgh School of Medicine Pittsburgh Pennsylvania USA; ^2^ Division of Health Services Research, Department of Urology University of Pittsburgh Medical Center Pittsburgh Pennsylvania USA; ^3^ Department of Health Policy and Management Graduate School of Public Health, University of Pittsburgh Pittsburgh Pennsylvania USA; ^4^ Center for Research on Heath Care Department of Medicine and Biostatistics, University of Pittsburgh Pittsburgh Pennsylvania USA; ^5^ Department of Urology Charleston Area Medical Center Charleston West Virginia USA; ^6^ Department of Critical Care Medicine, University of Pittsburgh School of Medicine Pittsburgh Pennsylvania USA; ^7^ University of Pittsburgh School of Medicine, UPMC Hillman Cancer Center Pittsburgh Pennsylvania USA; ^8^ Magee‐Women's Hospital Comprehensive Breast Program, Division of Surgical Oncology, University of Pittsburgh Medical Center Pittsburgh Pennsylvania USA; ^9^ Department of Neurosurgery University of Pittsburgh Medical Center Pittsburgh Pennsylvania USA; ^10^ Department of Obstetrics Gynecology and Reproductive Services, University of Pittsburgh School of Medicine, Magee‐Women's Hospital, University of Pittsburgh Medical Center Pittsburgh Pennsylvania USA; ^11^ Department of Cardiothoracic Surgery Wake Forest School of Medicine Winston Salem North Carolina USA; ^12^ Department of Epidemiology School of Public Health, University of Pittsburgh Pittsburgh Pennsylvania USA

**Keywords:** cancer surgery, centralization, readmissions, surgical disparities

## Abstract

**Background:**

While receipt of surgery at regional referral centers is associated with improved perioperative outcomes, many vulnerable patients may experience barriers in accessing these hospitals. When these patients do manage to undergo surgery at referral centers, it remains unclear where they are readmitted to receive care when complications arise. Patients may be readmitted to the hospital where surgery was performed (index readmission) or to a different hospital (non‐index readmission). This study examined whether factors associated with readmission to index versus non‐index hospitals differ for patients undergoing surgery at referral centers compared to non‐referral centers.

**Methods:**

We used data from the Pennsylvania Cancer Registry and the Pennsylvania Health Care Cost Containment Council (PHC4) to identify patients who had major cancer surgery and were subsequently readmitted within 90 days. We fit a multivariable logistic regression model to identify factors associated with 90 day readmission to an index versus non‐index hospital. We included an interaction term between referral center status and cancer type in this model.

**Results:**

A total of 8215 patients were readmitted within 90 days of cancer surgery, of whom 78% (*N* = 6388) were readmitted to the index hospital. On multivariable analysis, factors associated with lower odds of index versus non‐index readmission included older age, high Elixhauser comorbidity scores, and longer travel times. There was no significant difference in odds of index readmission when patients were treated at referral versus non‐referral centers (OR = 0.77; 95% CI, 0.50–1.20). When assessing interactions, patients with lung cancer had lower odds of index readmission when treated at referral versus non‐referral centers, relative to other cancers (OR = 0.59; 95% CI, 0.41–0.84).

**Conclusions:**

Higher clinical complexity and greater travel burdens were associated with lower odds of index readmission. Relative to other cancers, patients with lung cancer may be more likely to experience care fragmentation after undergoing surgery at a referral center.

## Graphical abstract

Older age, multiple comorbid conditions, and longer travel times were associated with lower odds of index readmissions. Referral center status yielded differing impacts by cancer type, with lung cancer patients experiencing lower odds of index readmission when treated at referral versus non‐referral centers.

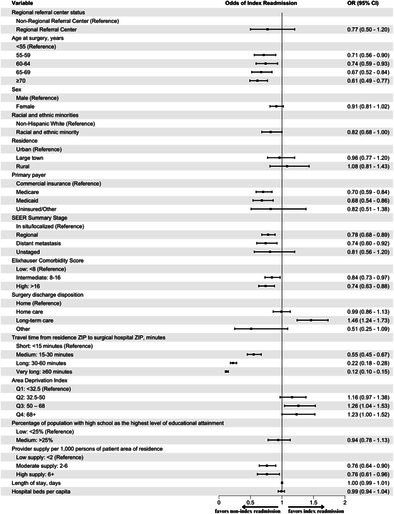

## 1 Introduction

Unplanned readmissions after cancer surgery occur in up to a quarter of cases and are associated with increased healthcare costs and mortality [1, 2]. Readmissions to the hospital where surgery was performed (index readmission) are associated with lower rates of mortality and postoperative complications compared with readmissions to a different hospital (non‐index readmission) [3]. Despite this, non‐index readmissions account for 20% of readmissions following cancer surgery [4]. These patterns have occurred in the context of increased centralization of cancer care, whereby patients requiring complex cancer surgeries are generally directed to high‐volume, regional referral centers [5].

Prior studies suggest that centralization is associated with improved perioperative outcomes [6, 7]. However, centralization presents a paradox: complex patients requiring advanced surgical care who stand to benefit the most from treatment at referral centers may face the greatest challenges in accessing these hospitals due in part to increased travel and transportation barriers [5, 8]. When these vulnerable patients do manage to undergo surgery at referral centers, it remains unclear where they are readmitted to receive postoperative care when unplanned complications arise.

To better understand this issue, we utilized state‐wide cancer registry data linked to an all‐payer, visit‐level inpatient database to analyze readmissions for patients who underwent major cancer surgery between 2013 and 2019. We examined the association between several patient‐, community‐, and hospital‐level factors and index versus non‐index readmissions, stratified by receipt of surgery at a referral versus a non‐referral center. To our knowledge, this is the first study that examines whether receipt of surgery at referral centers is associated with increased non‐index readmissions, thereby identifying a key opportunity to improve the quality of surgical cancer care delivery.

## 2 Methods

### 2.1 Study Population

In this retrospective study, we used data from the Pennsylvania Cancer Registry, which collects information on all newly diagnosed and treated cancer cases in Pennsylvania, and discharge records from the Pennsylvania Health Care Cost Containment Council (PHC4), an independent council that collects quarterly data on visits to all licensed health care facilities, covering approximately 1.7 million inpatient cases annually [9]. The Pennsylvania Cancer Registry has received gold certification by the North American Association of Central Cancer Registries for its high data quality [10].

We used International Classification of Diseases (ICD)‐9 and −10 procedure codes to identify major cancer surgeries in PHC4 and linked those hospital records to corresponding cancer diagnoses in the Pennsylvania Cancer Registry. We examined follow‐up data for all patients who underwent surgery between 2013 and 2019. We included adult patients with a cancer diagnosis within 1 year prior to surgery. We chose bladder, brain, esophageal, pancreatic, liver, and lung cancers because they are among the most lethal cancers and require complex, multidisciplinary treatments. These cancers also have readmission rates that rank among the highest of all cancer surgeries [1, 2, 11]. We used PHC4 to identify all discharges following major cancer surgery.

Next, we identified patients who had an unplanned readmission to a hospital in Pennsylvania within 90 days of their surgical discharge. We limited our study to the first readmission that occurred after surgery. The PHC4 dataset includes specific codes that indicate whether a patient was discharged with a scheduled plan for readmission. Because it is unclear whether the first readmission for these patients represented planned encounters or unexpected postoperative complications, we excluded these patients from our analysis. Planned readmission codes were exceedingly rare, applying to only 0.1% (*N* = 33) of patients in our dataset. For all other patients, we classified acute care hospital admissions after surgical discharge as unplanned readmissions [12]. Patients transferred between two acute care hospitals on the same day were not counted as a readmission, as these events likely reflect planned continuity of care.

### 2.2 Covariates

We identified whether cancer surgery occurred at any of the 16 regional referral centers in Pennsylvania. We defined regional referral centers as National Cancer Institute‐designated cancer centers or American College of Surgeons Commission on Cancer‐accredited academic comprehensive cancer programs [13].

We identified several patient‐ and community‐level covariates hypothesized to be associated with hospital readmission. Patient‐level covariates included age at surgery, race and ethnicity, sex, cancer type, year of surgery, primary payer, and surgery discharge disposition. We defined racial and ethnic minorities as non‐White and/or Hispanic. We did not further differentiate “non‐White” racial groups due to low patient counts in several subgroups. Race and ethnicity data were reported by the cancer registry at the time of diagnosis and included in this study because of their established association with cancer outcomes [14]. We defined patient residence as rural, large town, or urban using the United States Department of Agriculture Rural Urban Commuting Area Codes [15]. We evaluated patient comorbidities using the weighted Elixhauser Comorbidity Index, which is designed to predict the risk of 30 day, all‐cause readmission [16]. To provide an indication of the clinical stage of each patient's disease, we used the Surveillance, Epidemiology, and End Results (SEER) Summary Stage, a variable that classifies how far a cancer has spread from its point of origin [17]. To calculate travel time to the index hospital, we input the resident and hospital ZIP codes into the Google Maps Distance Matrix Application Programming Interface [18]. This program finds the best route between two ZIP code centroids using expected traffic conditions. We categorized travel time into four quartiles (< 15, 15–30, 30–60, and ≥ 60 min) based on an a priori review of expected travel times in the literature [19]. These categories were chosen based on distributions reported in prior studies and to ensure relatively balanced subgroup sizes [19].

We used the Area Deprivation Index (ADI) as a measure of neighborhood social disadvantage. ADI is a composite measure of 17 indicators of poverty, education, and housing evaluated at the United States Census tract level [20]. We reported ADI as quartiles, with the 4th quartile designated as most deprived. Other community‐level variables, including the percentage of a population with high school as the highest level of educational attainment, were captured at the Census tract level using data linked from the American Community Survey [21]. Provider supply and hospital beds per capita were measured at the county level using data linked from the Area Health Resources File [22].

### 2.3 Outcomes

Among our cohort of patients readmitted in the first 90 days after surgery, the primary outcome was whether the patient was readmitted to the index hospital versus a non‐index hospital. We defined an index readmission as a readmission to the same hospital where the original surgery took place and a non‐index readmission as one that occurred at any different acute care hospital. If a patient presented to a hospital postoperatively and was subsequently transferred, we attributed the readmission to the acute care hospital to which the patient was transferred. These events were rare occurrences, as less than 0.3% of patients (*N* = 22) experienced a readmission followed by a subsequent transfer. We ultimately chose this approach because we aimed to determine which of the two potential readmitting hospitals was more likely to provide definitive care.

### 2.4 Statistical Analysis

For each cancer type, we assessed 90 day readmission rates to index and non‐index hospitals. For the remainder of the analysis, we combined cancer types together to align with the methodology of prior studies [3, 4]. We used descriptive statistics to compare demographic characteristics of patients readmitted to index versus non‐index hospitals, stratified by whether surgery occurred at a referral versus a non‐referral center.

We fit a multivariable logistic regression model to identify which patient‐, community‐, and hospital‐level factors were associated with 90 day readmission to an index versus non‐index hospital. We also performed a sensitivity analysis using 30 day readmissions as the outcome. To examine the association between index readmission and surgery at a referral center among specific populations of interest, we included interaction terms between referral center status and (1) primary payer, (2) racial and ethnic minorities, (3) patient residence, (4) travel time, (5) ADI, and (6) cancer type. These variables have been previously associated with barriers to treatment at referral centers [8]. We first tested all interaction terms collectively using likelihood ratio tests, then refit the model by removing any interaction terms that were not significant. To address the issue of multiple hypothesis testing, we applied a false discovery rate adjustment using the Benjamini‐Hochberg procedure. Specifically, we extracted the *p*‐values from each likelihood ratio test comparing the full interaction model with a reduced model omitting one interaction term and then adjusted these *p*‐values for multiple comparisons. Only interaction terms with adjusted *p*‐values below the significance threshold were retained in the final model. We included one interaction term in the final model: the interaction between referral center status and cancer type.

To better understand the underlying reasons for the observed results among lung cancer patients, we conducted a post hoc sub‐analysis examining the surgical complexity of lung cancer patients readmitted to index and non‐index hospitals. We created a measure for surgical complexity by scoring the extent and invasiveness of each lung cancer procedure (Supporting Methods). We completed this analysis because of the significant differences in severity between lung cancer procedures (e.g., between wedge resection and a total pneumonectomy).

We performed data analyses in SAS v9.4 (SAS Institute Inc., Cary, NC) and R v4.2.0 (R Foundation for Statistical Computing, Vienna, Austria) [23]. All tests were two‐sided, and the probability of type I error was set at 0.05. The Institutional Review Board at the University of Pittsburgh deemed this study exempt from review.

## 3 Results

**FIGURE 1:**
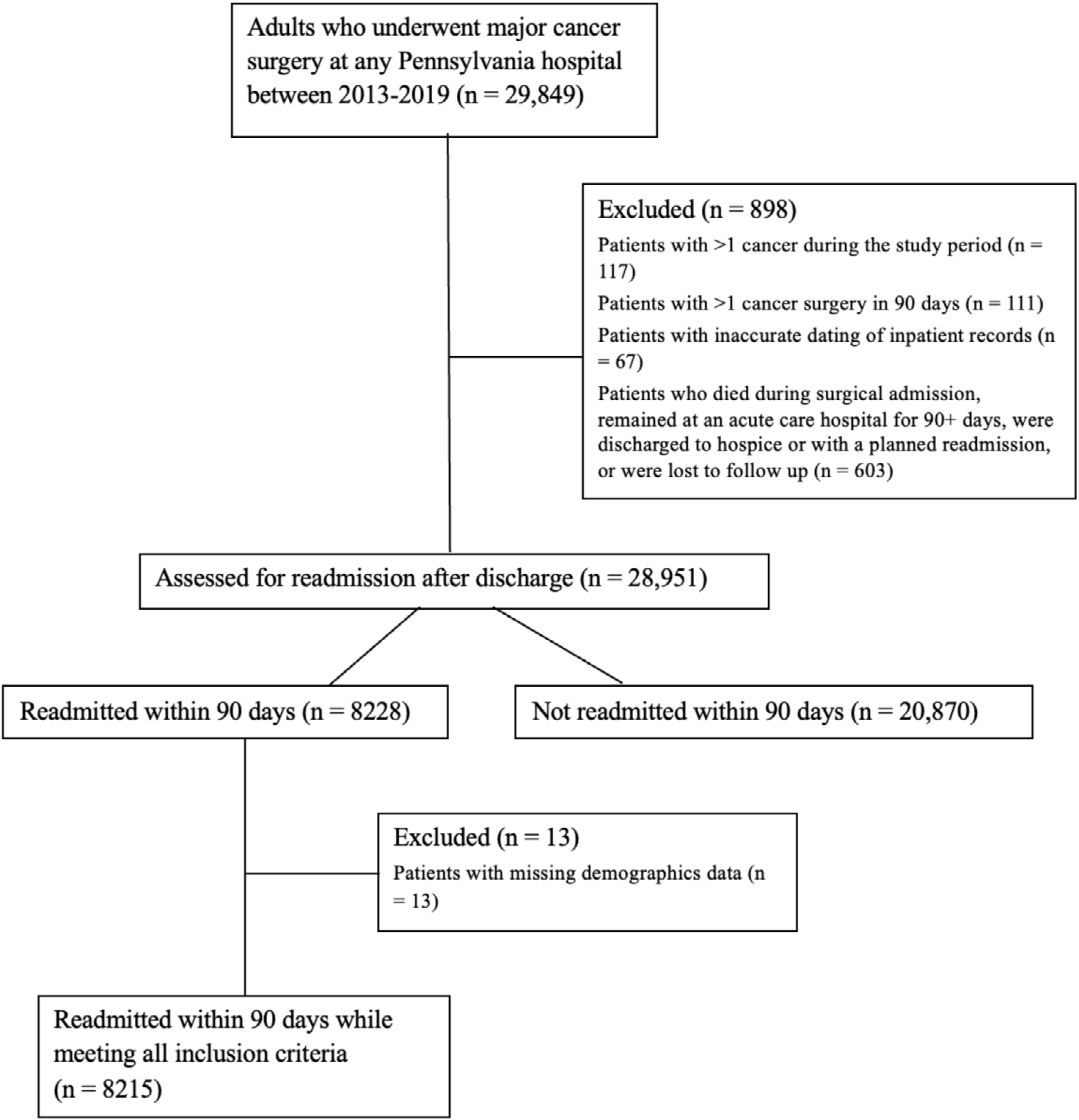
Patient selection and exclusion criteria.

We identified 28,951 patients with major cancer surgery who were discharged alive after surgery (Figure 1). A total of 8215 (28%) of these patients were readmitted within 90 days following surgery. Readmission rates for individual cancer types ranged from 21% for lung cancer to 44% for bladder cancer (Table 1). Of all patients readmitted, 57% (*N* = 4671) were originally treated at referral centers, and 78% (*N* = 6388) were readmitted to the index hospital.

**TABLE 1:** 30‐ and 90 day readmission rates by cancer type.

Cancer type	All	Bladder	Brain	Esophageal	Liver	Lung	Pancreatic
Number of patients discharged alive after cancer surgery	28,951	2364	7557	1047	809	13,815	3359
Number (%) of 30 day readmissions	5480 (19)	765 (32)	1867 (25)	240 (23)	152 (19)	1681 (12)	775 (23)
Number (%) of 90 day readmissions	8215 (28)	1051 (44)	2565 (34)	344 (33)	211 (26)	2867 (21)	1177 (35)

Among all readmitted patients, those treated at referral centers had lower rates of index readmissions compared to those treated at non‐referral centers (75% vs. 81%, *p* < 0.001) (Table 2). Among patients treated at referral centers, those with index readmissions were more often younger (age < 55: 20% vs. 11%, *p* < 0.001) and more likely to be racial and ethnic minorities (17% vs. 13%, *p* < 0.001) compared to those with non‐index readmissions.

**TABLE 2:** Patient, community, and hospital demographics by readmission type and referral center status.

Characteristic	Non‐regional referral center (*N* = 3544)			Regional referral center (*N* = 4671)		
	Index readmissions *N* = 2878 (81%)	Non‐index readmissions *N* = 666 (19%)	*p*	Index readmissions *N* = 3510 (75%)	Non‐index readmissions *N* = 1161 (25%)	*p*
Age at surgery, years, *N* (%)			0.23			< 0.001
< 55	385 (85)	67 (15)		704 (84)	132 (16)	
55–59	301 (80)	73 (20)		390 (77)	113 (23)	
60–64	397 (80)	98 (20)		510 (79)	135 (21)	
65–69	501 (81)	115 (19)		606 (72)	237 (28)	
≥ 70	1294 (81)	313 (19)		1300 (70)	544 (30)	
Sex, *N* (%)			0.99			0.95
Male	1654 (81)	382 (19)		2002 (75)	664 (25)	
Female	1224 (81)	284 (19)		1508 (75)	497 (25)	
Racial and ethnic minorities, *N* (%)			0.52			< 0.001
Non‐Hispanic white	2642 (81)	617 (19)		2910 (74)	1015 (26)	
Racial and ethnic minority^a^	236 (83)	49 (17)		600 (80)	146 (20)	
Cancer type, *N* (%)			0.67			< 0.001
Bladder	377 (82)	81 (18)		419 (71)	174 (29)	
Brain	738 (82)	164 (18)		1397 (84)	266 (16)	
Esophageal	97 (80)	24 (20)		169 (76)	54 (24)	
Liver	> 49 (> 82)	< 11 (< 18)		112 (74)	39 (26)	
Lung	< 1338 (< 80)	> 329 (> 20)		786 (65)	414 (35)	
Pancreatic	279 (83)	57 (17)		627 (75)	214 (25)	
Surgery year, *N* (%)			0.80			0.50
2013	452 (81)	107 (19)		520 (75)	169 (25)	
2014	434 (80)	108 (20)		481 (75)	164 (25)	
2015	448 (82)	99 (18)		494 (74)	173 (26)	
2016	432 (83)	89 (17)		518 (78)	145 (22)	
2017	398 (80)	103 (20)		520 (75)	177 (25)	
2018	355 (81)	83 (19)		493 (73)	180 (27)	
2019	359 (82)	77 (18)		484 (76)	153 (24)	
Residence, *N* (%)			< 0.001			0.001
Urban	2432 (83)	511 (17)		3222 (76)	1025 (24)	
Large town	311 (75)	102 (25)		188 (68)	87 (32)	
Rural	135 (72)	53 (28)		100 (67)	49 (33)	
Primary payer, *N* (%)			0.11			< 0.001
Commercial insurance	768 (83)	157 (17)		1241 (82)	276 (18)	
Medicare	1798 (80)	> 443 (> 20)		1895 (71)	781 (29)	
Medicaid	268 (83)	55 (17)		323 (79)	88 (21)	
Uninsured/Other^b^	44 (80)	< 11 (< 20)		51 (76)	16 (24)	
SEER summary stage, *N* (%)			0.003			< 0.001
In situ/localized	1610 (83)	> 325 (> 17)		1936 (80)	498 (20)	
Regional	1019 (79)	264 (21)		1230 (70)	535 (30)	
Distant metastasis	200 (75)	66 (25)		230 (70)	99 (30)	
Unstaged	49 (82)	< 11 (< 18)		114 (80)	29 (20)	
Elixhauser Comorbidity Score, *N* (%)			0.39			< 0.001
Low: < 8	637 (83)	134 (17)		962 (80)	238 (19)	
Intermediate: 8–16	1521 (81)	352 (19)		1778 (74)	626 (26)	
High: > 16	720 (80)	180 (20)		770 (72)	297 (28)	
Surgery discharge disposition, *N* (%)			< 0.001			< 0.001
Home	914 (79)	238 (21)		976 (75)	329 (25)	
Home care	975 (80)	248 (20)		1266 (70)	530 (30)	
Long‐term care^c^	971 (85)	169 (15)		> 1257 (> 81)	> 291 (> 19)	
Other^d^	18 (62)	11 (38)		< 11 (50)	< 11 (50)	
Travel time from residence ZIP to surgical hospital ZIP, minutes, *N* (%)			< 0.001			< 0.001
Short: < 15 min	839 (91)	85 (9)		748 (88)	102 (12)	
Medium: 15–30 min	1134 (86)	183 (14)		1009 (82)	225 (18)	
Long: 30–60 min	652 (73)	235 (27)		1140 (71)	469 (29)	
Very Long: ≥ 60 min	253 (61)	163 (39)		613 (63)	365 (37)	
Area Deprivation Index, *N* (%)			0.01			0.007
Q1: < 32.5	531 (85)	97 (15)		1023 (72)	394 (28)	
Q2: 32.5–50	800 (82)	171 (18)		825 (75)	275 (25)	
Q3: 50–68	789 (81)	189 (19)		807 (76)	250 (24)	
Q4: 68+	758 (78)	209 (22)		855 (78)	242 (22)	
Percentage of population with high school as the highest level of educational attainment, *N* (%)			0.13			0.23
Low: < 25%	297 (84)	55 (16)		827 (77)	253 (23)	
Medium: > 25%	2581 (81)	611 (19)		2683 (75)	908 (25)	
Provider supply per 1000 persons of patient area of residence, *N* (%)			< 0.001			< 0.001
Low supply: < 2	793 (74)	268 (26)		697 (73)	253 (27)	
Moderate supply: 2–6	1661 (84)	320 (16)		1591 (72)	629 (28)	
High supply: ≥ 6	424 (85)	78 (15)		1222 (81)	279 (19)	
Length of stay of surgical admission, days, Median (IQR)^e^	7 (6)	6 (6)	0.006	7 (6)	6 (5)	0.001
Hospital beds per capita of patient's area of residence, Median (IQR)^e^	2.7 (1.6)	2.7 (1.7)	0.52	2.6 (2)	2.3 (2)	< 0.001

*Note:* Row percentages may not add to 100 due to rounding. In compliance with the PHC4 Data Use Agreement, all cells with *N* < 11 are not reported. Chi‐square tests are used for categorical variables, and *t*‐tests are used for continuous variables to determine *p*‐values.

^a^ Racial and ethnic minorities: racial and ethnic minorities are defined as patients who were non‐white and/or Hispanic.

^b^ Primary payer: Other defined as self‐pay, government, or missing.

^c^ Discharge disposition: long‐term care defined as skilled nursing facilities, long‐term acute care hospitals, inpatient rehabilitation, and Medicare swing bed.

^d^ Discharge disposition: Other defined as government health facilities, undefined health care institutions, and patients who left against medical advice.

^e^ IQR is an abbreviation for interquartile range.

**FIGURE 2:**
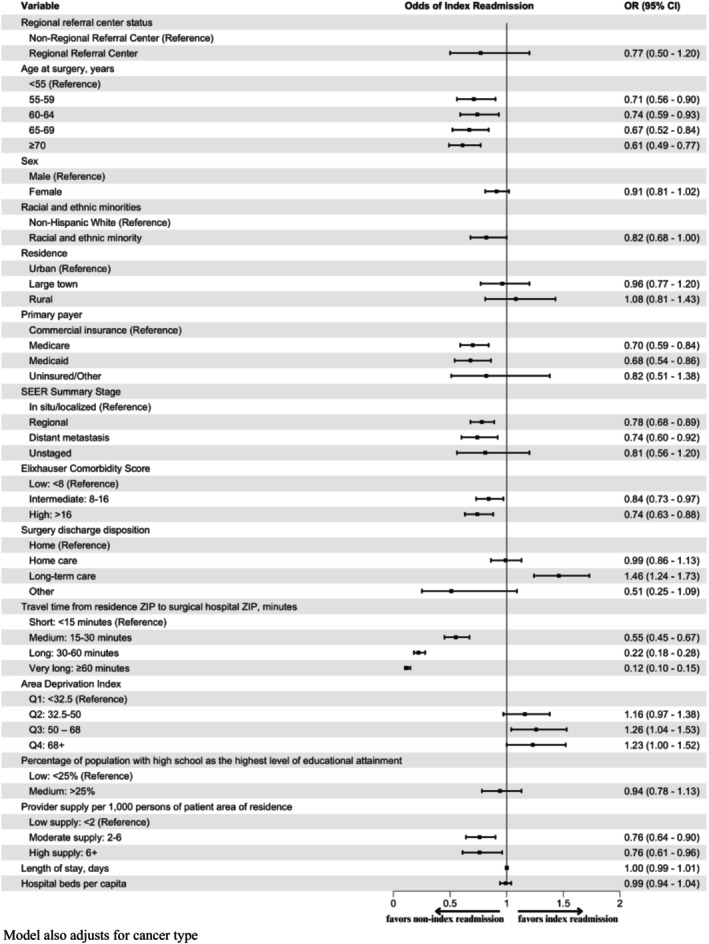
Multivariable logistic regression for factors associated with 90 day readmission to index versus non‐index hospitals.

On multivariable analysis, factors associated with lower odds of index versus non‐index readmission included older age (≥ 70 years: odds ratio (OR) = 0.61; 95% confidence interval (CI), 0.49–0.77, compared to < 55 years), high Elixhauser comorbidity scores (> 16: OR = 0.74; 95% CI, 0.63–0.88; compared to < 8), and longer travel times (> 60 min: OR = 0.12; 95% CI, 0.10–0.15; compared to < 15 min), among others (Figure 2). Discharge to a long‐term care facility after surgery was associated with higher odds of an index readmission compared to discharge home (OR = 1.46; 95% CI, 1.24–1.73). On 30 day sensitivity analysis, point estimates for factors associated with index versus non‐index readmission were largely unchanged, though some confidence intervals were wider in the 30 day analysis (Table S1 & S2). In particular, the association between age and readmission location appeared less significant at 30 days compared to 90 days (65–69 years vs. < 55 years: OR = 0.74, 95% CI: 0.52–1.03 at 30 days; OR = 0.67, 95% CI: 0.52–0.84 at 90 days).

**FIGURE 3:**
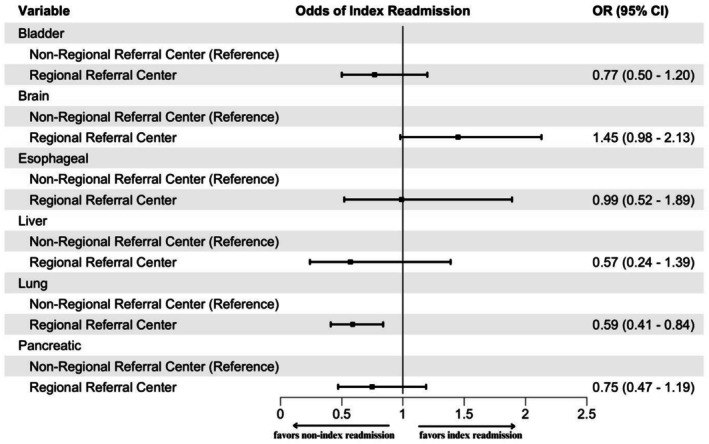
Interaction between referral center status and cancer type from multivariable logistic regression.

When assessing interactions, patients with lung cancer treated at a referral center had 41% lower odds of index readmission compared to those treated at a non‐referral center, relative to other cancers (OR = 0.59; 95% CI, 0.41–0.84) (Figure 3). For patients with lung cancer treated at referral centers, there was a statistically significant association between surgery complexity and index versus non‐index readmission rates, with higher complexity surgeries associated with more frequent non‐index readmissions (*p* = 0.001) (Table S3).

## 4 Discussion

In this study, we found that nearly one‐quarter of readmissions after major cancer surgery were to non‐index hospitals. There was no significant difference in overall odds of index readmission for patients treated at referral versus non‐referral centers. Dissimilar to other cancers, the odds of index readmission were significantly lower for lung cancer patients who were treated at referral versus non‐referral centers. We identified several factors associated with decreased odds of index readmission compared to non‐index readmission, including older age, increased comorbidities, and increased travel time.

Lung cancer was the only cancer type associated with significantly lower odds of index readmission when treated at referral versus non‐referral centers. One possible explanation is that providers at non‐referral centers may be more comfortable managing postoperative pulmonary complications. Several of these complications, including pneumonia, pleural effusion, and pneumothorax, are common general emergency presentations that are regularly managed without the specialized care of a referral center [24]. In our results, the interaction between liver cancer and referral center status showed an odds ratio similar to that of lung cancer, though with a wider confidence interval. After hepatectomy, pulmonary complications are exceedingly common, with around 35% of patients experiencing pleural effusion and 10% of patients experiencing pneumonia [25]. This overlap in pulmonary complications may partially explain the similarity in effect estimates. Additionally, lung cancer surgery encompasses a range of procedures—including wedge resections, lobectomies, and pneumonectomies—that differ in associated risks and mortality [26]. Our results show that the varying complexity of these lung cancer procedures may be contributing toward differences in index and non‐index readmission rates.

Among all factors analyzed in this study, travel time emerged as the strongest factor associated with non‐index readmission. Readmitted patients residing more than an hour from the index hospital had 88% lower odds of returning to the index hospital. These findings are consistent with prior studies demonstrating that patients living greater distances away from the index hospital are more likely to utilize local emergency departments once discharged [1]. Postoperative care for major cancer surgeries often involves frequent follow‐up with the surgical team. However, patients with longer travel distances may miss or delay appointments, increasing the likelihood that complications intensify before they are addressed. Prior work has shown that patients who require readmission for urgent complications are less likely to return to the index hospital compared to patients with routine readmissions [4]. Thus, the strong association between travel time and readmission location may reflect not only inconvenience from distance but also the role of travel time acting as a surrogate for perceived complication urgency. Over recent years, as fewer hospitals are performing major cancer surgeries, patient travel times have increased [27]. Our results show that while patients may be willing to spend more time traveling for planned surgeries, they are more likely to seek care that requires less travel for unplanned encounters.

Consistent with our findings, previous studies have demonstrated that advanced age and higher comorbidity scores are associated with decreased rates of index readmissions [3, 4]. Longer travel times may be particularly burdensome for these patient groups after surgery. Older adults and patients with comorbidities likely experience greater postoperative functional limitations, making transportation a significant barrier [28]. As a result, these individuals may be physically limited in seeking care locally rather than traveling back to the index hospital. Furthermore, these patients may face an increased risk of higher acuity, medical complications, such as myocardial infarction or stroke, after surgery. Evidence suggests that patients experiencing medical complications are more likely to be readmitted to non‐index hospitals than those with surgical complications [3, 29]. Social isolation and cognitive impairment, common in aging, highlight the need for care continuity in this population [30]. However, our findings reveal that these complex patients are more frequently readmitted to non‐index hospitals after cancer surgery, where they remain subject to the challenges of care fragmentation.

Our study results have several policy implications. For lung cancer patients treated at referral centers, strategies to improve care coordination may include involving the local referring physician in post‐discharge planning and encouraging earlier outpatient follow‐up, which has been shown to decrease readmission rates for lower‐acuity complications [31]. Patients with longer travel times to the index hospital may benefit from measures such as non‐emergency medical transportation assistance and the expanded use of telemedicine to triage postoperative complications [28]. Finally, older patients and patients with increased clinical complexity could be supported through timely inter‐hospital transfers once patients are stabilized and the more liberal use of post‐acute care facilities upon discharge.

These findings should be interpreted in the context of several limitations. First, using PHC4 data may limit the generalizability of this study to the rest of the United States. However, the PHC4 dataset serves as a strong model for national outcomes, as Pennsylvania has demographic characteristics similar to other states (18% racial and ethnic minority population, 26% rural residents) and has the fifth‐highest incidence of new cancers [32, 33]. Second, our dataset is limited in its ability to capture admissions to hospitals outside of Pennsylvania. Thus, it is possible that we missed patients who had surgery in Pennsylvania but were subsequently readmitted to hospitals in other states. Despite this, our results are consistent with previously reported readmission rates by cancer type, suggesting that this limitation likely had little effect on our findings. Third, we lack data on individual‐level variables for socioeconomic status, and instead rely on community‐level covariates such as ADI. Although not ideal, ADI has been previously validated for examining post‐surgical readmission disparities when individual‐level data are unavailable [34]. Fourth, the PHC4 dataset does not provide detailed information about initial emergency department presentations unless they led to an inpatient admission or transfer. Therefore, we were unable to map the entire care‐seeking pathway, including ED‐only visits without admissions, in this study. Fifth, we used 90 day follow‐up for our primary outcome, which may be more influenced by disease course and comorbidities rather than short‐term surgical complications. However, our 30 day sensitivity analyses were largely consistent with the primary results. While we did observe some differences in the association between age and readmission location at 30 versus 90 days, these findings may reflect greater statistical uncertainty due to fewer readmission events within the shorter timeframe. Sixth, although index readmissions have been previously associated with improved outcomes, it remains unclear whether this is attributable solely to improved care coordination or if there is added selection bias favoring patients with less urgent complications. In our analysis, we identified several factors independently associated with index versus non‐index readmission, including travel time, surgical complexity, age, and comorbidity scores. However, these factors may also serve as proxies for postoperative acuity, complicating overall interpretation.

Despite these limitations, our study makes important contributions to evidence on postoperative care for the following reasons. This analysis builds on prior studies of readmission patterns after major cancer surgery by introducing a novel variable for receipt of surgery at a referral center [3, 35]. By linking community data with statewide hospital‐ and patient‐level data, we found that several factors, including older age, comorbid conditions, and longer travel times, were associated with lower rates of index readmissions. Patients with lung cancer had lower odds of index readmission when treated at referral centers compared to non‐referral centers. Overall, this pattern suggests that the association between referral center status and readmission location may vary based on complication type and surgical complexity.

## Author Contributions

**Avanish Madhavaram:** conceptualization (equal), formal analysis (equal), investigation (equal), methodology (equal), writing – original draft (equal), writing – review and editing (equal). **Shan Wu:** data curation (equal), formal analysis (equal), investigation (equal), methodology (equal), writing – original draft (equal), writing – review and editing (equal). **Danielle R. Sharbaugh:** data curation (equal), formal analysis (equal), methodology (equal), writing – review and editing (equal). **Yuanbo Zhang:** data curation (equal), formal analysis (equal), writing – review and editing (equal). **Jonathan G. Yabes:** conceptualization (equal), investigation (equal), methodology (equal), supervision (equal), writing – review and editing (equal). **Kathryn A. Marchetti:** conceptualization (equal), investigation (equal), methodology (equal), writing – review and editing (equal). **Michael G. Stencel:** methodology (equal), writing – review and editing (equal). **Kimberly J. Rak:** conceptualization (equal), investigation (equal), methodology (equal), writing – review and editing (equal). **John A. Lech:** conceptualization (equal), investigation (equal), writing – review and editing (equal). **Emilia J. Diego:** conceptualization (equal), investigation (equal), writing – review and editing (equal). **Pascal O. Zinn:** conceptualization (equal), writing – review and editing (equal). **Sarah Taylor:** conceptualization (equal), investigation (equal), writing – review and editing (equal), writing – review and editing (equal). **Rajeev Dhupar:** conceptualization (equal), writing – review and editing (equal). **Dana H. Bovbjerg:** conceptualization (equal), investigation (equal), supervision (equal), writing – review and editing (equal). **Tiffany L. Gary‐Webb:** conceptualization (equal), investigation (equal), supervision (equal), writing – review and editing (equal). **Jeremy M. Kahn:** conceptualization (equal), investigation (equal), methodology (equal), supervision (equal), writing – review and editing (equal). **Lindsay M. Sabik:** conceptualization (equal), investigation (equal), methodology (equal), resources (equal), supervision (equal), writing – review and editing (equal). **Bruce L. Jacobs:** conceptualization (equal), formal analysis (equal), funding acquisition (equal), investigation (equal), methodology (equal), resources (equal), supervision (equal), writing – original draft (equal), writing – review and editing (equal).

## Ethics Statement

The Institutional Review Board (IRB) at the University of Pittsburgh deemed this study exempt from review. This IRB considered this study exempt from written informed consent due to the use of secondary, deidentified data.

## Conflicts of Interest

The authors declare no conflicts of interest.

## Supporting information


**Table S1:** 30‐day sensitivity analysis: Multivariable logistic regression for factors associated with 30‐day readmission to index versus non‐index hospitals.
**Table S2:** 30‐day sensitivity analysis: Interactions term from multivariable logistic regression model.
**Table S3:** Readmission location by surgery complexity for patients with lung cancer only.

## Data Availability

The data that support the findings of this study are available from the Bureau of Health Statistics & Registries, Pennsylvania Department of Health, Harrisburg, Pennsylvania and the Pennsylvania Health Care Cost Containment Council. Restrictions apply to the availability of these data, which were used under permission for this study.
