# Supplementary material for: Factors Associated With Readmission to Index vs. Non‐Index Hospitals After Major Cancer Surgery
Source: Cancer Med. 2025 Dec 17;14(24):e71359. doi: 10.1002/cam4.71359 (PMC12712393; doi:10.1002/cam4.71359)
Supplement: Supplementary file 1 — Table S1: 30‐day sensitivity analysis: Multivariable logistic regression for factors associated with 30‐day readmission to index versus non‐index hospitals. Table S2: 30‐day sensitivity analysis: Interactions term from multivariable logistic regression model. Table S3: Readmission location by surgery complexity for patients with lung cancer only. [file CAM4-14-e71359-s001.docx]

Supplemental Methods. Measuring surgery complexity for lung cancer procedures

Surgeries for lung cancer are numerous, and the expected peri-operative morbidity is variable and unique to each procedure. We created a novel scoring system to quantify the relative expected morbidity using existing literature.

We assigned a score of 1-5 (least to most complex) to each ICD-9/10 procedure and CPT code based on extent of resection, invasiveness of resection, and surgical approach.^1-3^ Each score was derived by first considering the extent of resection (pneumonectomy>lobectomy>bronchus), where a more extensive resection earned a higher relative score. Then, each procedure code was given an additional point if the procedure code included the term “resection” (ie. removal of an entire organ) and was considered more complex than a partial excision. Finally, we considered an “endoscopic” approach less complex and subtracted one point for these codes. The final score was then used as a categorical variable to compare index versus non-index readmission rates.

References

1. Strand TE, Rostad H, Damhuis RAM, Norstein J. Risk factors for 30-day mortality after resection of lung cancer and prediction of their magnitude. Thorax. 2007 Nov;62(11):991–7.

2. Phillips JD, Merkow RP, Sherman KL, DeCamp MM, Bentrem DJ, Bilimoria KY. Factors affecting selection of operative approach and subsequent short-term outcomes after anatomic resection for lung cancer. J Am Coll Surg. 2012 Aug;215(2):206–15.

3. Desai H, Natt B, Kim S, Bime C. Decreased In-Hospital Mortality after Lobectomy Using Video-assisted Thoracoscopic Surgery Compared with Open Thoracotomy. Ann Am Thorac Soc. 2017 Feb;14(2):262–6.

Supplemental Table 1. 30-day sensitivity analysis: Multivariable logistic regression for factors associated with 30-day readmission to index versus non-index hospitals

| **Variable** | **Odds of Index Readmission, OR (95% CI)** |
| --- | --- |
| **Age at surgery, years** |  |
| < 55 | 1 (reference) |
| 55-59 | 0.76 (0.54, 1.06) |
| 60-64 | 0.87 (0.63, 1.20) |
| 65-69 | 0.74 (0.52, 1.03) |
| ≥ 70 | 0.65 (0.47, 0.90) |
| **Sex** |  |
| Male | 1 (reference) |
| Female | 0.98 (0.83, 1.15) |
| **Racial and ethnic minorities** |  |
| Non-White Hispanic | 1 (reference) |
| Racial and ethnic minority^a^ | 0.76 (0.58, 0.99) |
| **Residence** |  |
| Urban | 1 (reference) |
| Large town | 0.99 (0.73, 1.35) |
| Rural | 0.98 (0.68, 1.45) |
| **Primary payer** |  |
| Commercial insurance | 1 (reference) |
| Medicare | 0.66 (0.51, 0.85) |
| Medicaid | 0.75 (0.54, 1.04) |
| Uninsured/Other^b^ | 0.69 (0.37, 1.39) |
| **SEER Summary Stage** |  |
| In situ/localized | 1 (reference) |
| Regional | 0.88 (0.73, 1.06) |
| Distant Metastasis | 0.60 (0.45, 0.80) |
| Unstaged | 1.20 (0.67, 2.35) |
| **Elixhauser Comorbidity Score** |  |
| Low (<8) | 1 (reference) |
| Intermediate (8-16) | 0.82 (0.67, 1.01) |
| High (>16) | 0.76 (0.60, 0.96) |
| **Surgery discharge disposition** |  |
| Home | 1 (reference) |
| Home Care | 1.08 (0.88, 1.32) |
| Long Term Care^c^ | 1.88 (1.49, 2.37) |
| Other^d^ | 0.78 (0.32, 2.15) |

| **Variable** | **Odds of Index Readmission, OR (95% CI)** |
| --- | --- |
| **Travel time from residence ZIP to surgical hospital ZIP, minutes** |  |
| Short <15 minutes | 1 (reference) |
| Medium 15-30 minutes | 0.68 (0.52, 0.89) |
| Long 30-60 minutes | 0.28 (0.21, 0.37) |
| Very Long ≥60 minutes | 0.17 (0.12, 0.22) |
| **Area Deprivation Index** |  |
| Q1: <32.5 | 1 (reference) |
| Q2: 32.5 -50 | 1.20 (0.94, 1.55) |
| Q3: 50 – 68 | 1.14 (0.87, 1.49) |
| Q4: 68+ | 1.27 (0.95, 1.69) |
| **Percentage of population with high school as the highest level of educational attainment, N(%)** |  |
| Low (<25%) | 1 (reference) |
| Medium (25-75%) | 0.83 (0.63, 1.08) |
| **Provider supply per 1,000 persons of patient area of residence, N(%)** |  |
| Low supply: <2 | 1 (reference) |
| Moderate supply: 2-6 | 0.70 (0.55, 0.89) |
| High supply: ≥6 | 0.71 (0.51, 0.99) |
| **Length of stay, days** | 0.99 (0.98, 1.00) |
| **Hospital beds per capita** | 0.98 (0.92, 1.06) |

^a^ Racial and ethnic minorities: racial and ethnic minorities defined as patients who were non-white and/or Hispanic

^b^ Primary payer: other defined as self-pay, government, or missing

^c^ Discharge disposition: long-term care defined as skilled nursing facilities, long-term acute care hospitals, inpatient rehabilitation, and Medicare swing bed

^d^ Discharge disposition: other defined as government health facilities, undefined health care institutions, and patients who left against medical advice

Supplemental Table 2. 30-day sensitivity analysis: Interactions term from multivariable logistic regression model

| **Variable** | **Odds of Index Readmission, OR (95% CI)** |
| --- | --- |
| **Cancer Site** |  |
| **Bladder** |  |
| Regional Referral Center | 1 (reference) |
| Non-Regional Referral Center | 0.80 (0.54, 1.20) |
| **Brain** |  |
| Regional Referral Center | 1 (reference) |
| Non-Regional Referral Center | 1.50 (1.09, 2.06) |
| **Esophageal** |  |
| Regional Referral Center | 1 (reference) |
| Non-Regional Referral Center | 1.27 (0.60, 2.68) |
| **Liver** |  |
| Regional Referral Center | 1 (reference) |
| Non-Regional Referral Center | 0.42 (0.11, 1.54) |
| **Lung** |  |
| Regional Referral Center | 1 (reference) |
| Non-Regional Referral Center | 0.55 (0.42, 0.71) |
| **Pancreatic** |  |
| Regional Referral Center | 1 (reference) |
| Non-Regional Referral Center | 0.95 (0.56, 1.60) |

Supplemental Table 3. Readmission location by surgery complexity for patients with lung cancer only

|  | **Non-Regional Referral Center (N=1200)** | | | **Regional Referral Center (N=1667)** | | |
| --- | --- | --- | --- | --- | --- | --- |
| **Characteristic** | **Index readmissions N=1336 (80%)** | **Non-Index readmissions N=331 (20%)** | **P Value** | **Index readmissions**  **N= 786 (66%)** | **Non-Index readmissions N=414 (34%)** | **P Value** |
| **Surgery complexity, N (%)** |  |  | 0.02 |  |  | 0.001 |
| 1 (least complex) | 35 (3) | 14 (4) |  | 34 (4) | 33 (8) |  |
| 2 | 330 (25) | 75 (23) |  | 200 (25) | 77 (19) |  |
| 3 | 542 (41) | >151 (>46) |  | 333 (42) | >162 (>39) |  |
| 4 | 417 (31) | 80 (24) |  | 203 (26) | 131 (32) |  |
| 5 (most complex) | 12 (1) | <11 (<3) |  | 16 (2) | <11 (<3) |  |

Column percentages may not add to 100 due to rounding

In compliance with the PHC4 Data Use Agreement, all cells with N<11 are not reported

Chi-square tests used to determine p-values
